# The most responsive foot position for non-invasive detection of isolated unstable syndesmotic injuries – a 3D analysis

**DOI:** 10.1186/s13018-024-05211-y

**Published:** 2024-11-02

**Authors:** Firas Souleiman, Martin Heilemann, Georg Osterhoff, Pierre Hepp, Boyko Gueorguiev, R. Geoff Richards, Dominic Gehweiler, Robert Hennings

**Affiliations:** 1 AO Research Institute Davos, Davos, Switzerland; 2 Department of Orthopedics, Trauma and Plastic Surgery, University of Leipzig, Leipzig, Germany; 3 ZESBO - Center for Research on Musculoskeletal Systems, Leipzig University, Leipzig, Germany

**Keywords:** Syndesmosis, 3D measurement, syndesmotic injury, computed tomography, weightbearing, foot position

## Abstract

**Background:**

The aim of this study was to identify the most responsive foot position for detection of isolated unstable syndesmotic injury.

**Methods:**

Fourteen paired human cadaveric lower legs were positioned in a pressure-controlled radiolucent frame and loaded under 700 N. Computed tomography scans were performed in neutral position, 15° internal / external rotation, and 20° dorsal / plantar flexion of the foot before and after cutting all syndesmotic ligaments. For each position, generated 3D models of the intact and injured distal tibiofibular joints were matched and analyzed by calculating three parameters: diastasis, anteroposterior displacement, and shortening of the fibula.

**Results:**

Transection of syndesmotic ligaments resulted in significant posterior translation of the fibula (4.34°, SD 1.63°, *p* < 0.01) compared to uninjured state for external rotation, significant anterior translation (-2.08°, SD 1.65°, *p* < 0.01) for internal rotation, and significant posterior translation (1.32°, SD 1.16°, *p* = 0.01) for dorsiflexion. Furthermore, the syndesmotic injury led to significantly increased clear space (0.46 mm, SD 0.46 mm, *p* = 0.03) in external rotation of the foot.

**Conclusion:**

External rotation of the foot under loading seems to be the most responsive position for detection of isolated syndesmotic instability. Under external rotational stress, anteroposterior instability and increased clear space resulting from a complete isolated unstable syndesmotic lesion were most evident.

## Background

Ankle sprains are among the most common injuries during sport activities and reasons for consultations in emergency departments. Isolated injuries of the distal tibiofibular joint (DTFJ) – i.e. without a fracture of the ankle – occur in up to 30% of ankle sprains and are often missed or delayed in diagnosis [1–3]. Furthermore, they are more likely to result in long-term dysfunction and require much longer time for recovery [1, 4, 5]. Due to pain, the immediate posttraumatic examination is challenging and limited in assessment [4]. 

The syndesmotic complex consists of three major ligaments centering the distal fibula in the tibial incisura by three point stabilization – the anterior inferior tibiofibular ligament (AiTFL), the interosseous membrane/ligament (IOL), and the posterior inferior tibiofibular ligament (PiTFL) [6]. The modified West Point Ankle Grading System is commonly used and grades in terms of ligamentous injury severity [7]. It provides a differentiation between stable (Grade I/ IIA – sprain/rupture of the AiTFL) and unstable (Grade IIB/ III – rupture of AiTFL, IOL/plus PiTFL) syndesmosis injuries [8]. Up to date, the differentiation between stable and unstable situations requiring surgical stabilization remains clinically challenging.

Several tests, such as the Frick external rotation-, the squeeze-, the fibula translation-, and the Cotton-test exist for clinical examination [7, 9, 10]. However, none of them is sufficiently reliable or accurate to detect isolated syndesmotic injuries with reasonable certainty [10–12]. Mostly, the treatment choice is based on widening of the tibiofibular clear space under external rotational stress seeing under fluoroscopy [13, 14]. However, radiographic or fluoroscopic examinations depend on the position or rotation of the ankle joint, the image quality, and the examiner [15–18]. An ideal radiographic parameter for assessment of a syndesmotic injury does not exist [16]. Therefore, additional diagnostic procedures such as magnetic resonance imaging (MRI), and weightbearing cone-beam computed tomography (WBCT) are recommended, being more sensitive and accurate than radiographs in predicting syndesmotic injuries [17–21]. MRI is considered as the noninvasive gold standard, but has its limitations in the visualization and evaluation of the IOL at the level of the syndesmotic complex. Additionally, all these imaging techniques are static and do not objectify the dynamic aspect of instability due to the anatomy [7]. 

The foot position during a trauma accident is relevant for the pathomechanism and morphology of ligament rupture or fracture (e.g. forced external rotation with excessive dorsiflexion) [22]. 

Current studies demonstrated that the position of the foot affects the configuration of DTFJ of both uninjured and injured ankles during CT imaging [23–26]. There is no consistent recommendation for the foot position during imaging for a reliable detection of unstable syndesmotic lesions [7]. Despite the known impact of foot position on DTFJ configuration, cross-sectional imaging (CT, MRI) is performed in neutral position of the foot.

Therefore, the aim of this study was to determine the most responsive foot position for non-invasive detection of isolated unstable syndesmotic injuries via automated three-dimensional (3D) analysis.

## Methods

This study was performed in line with the principles of the Declaration of Helsinki All donors gave their informed consent inherent within the donation of the anatomical gift statement during their lifetime.

**Fig. 1:**
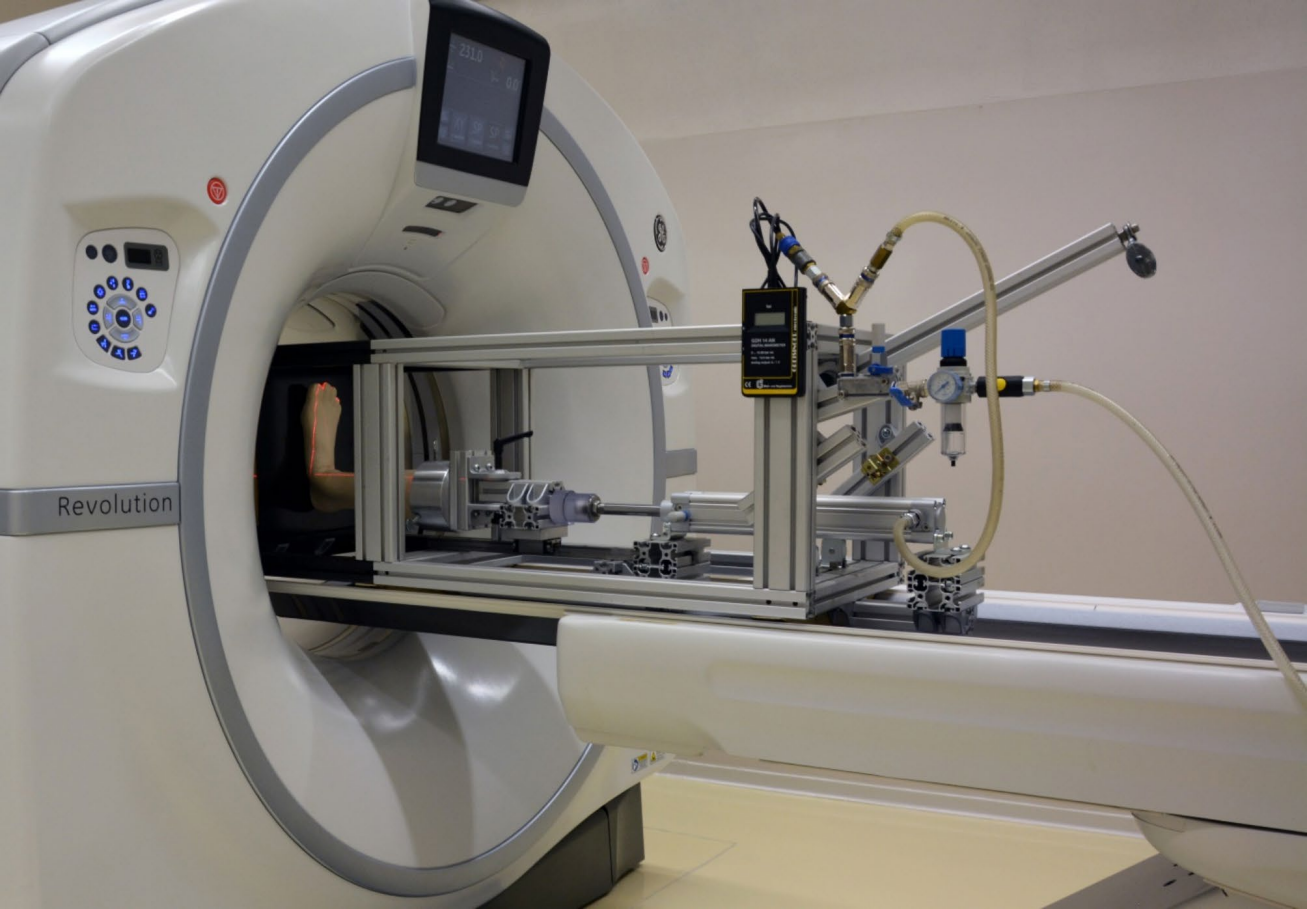
Custom-made loading frame with an artificial specimen mounted for CT scanning under weightbearing. The distal end of the frame is made out of radiolucent composite material, the main part – out of aluminum. A pneumatic cylinder is connected to a compressed air system at the proximal end of the frame for specimen’s loading

Fourteen paired fresh-frozen (− 20 °C) human cadaveric lower legs from 4 male and 3 female donors aged 81 years on average (range 59 to 91 years) were examined. The specimens revealed no signs of preexisting pathology, trauma, or surgery. The specimens were thawed 24 h before preparation, then cut and embedded at the level of the middle tibia below the tuberosity in polymethylmethacrylate (PMMA; SCS-Beracryl, Suter-Kunststofe AG, Fraubrunnen, Switzerland) with intact syndesmotic ligaments and interosseous membrane. The fibula was dissected at the level of embedding to be excluded from fixation [23, 27–29]. Each specimen was mounted horizontally in an air pressure-controlled radiolucent frame, specifically designed for positioning and axial loading of human cadaveric lower legs during CT scanning (Fig. 1).

All 14 ankle joints demonstrated an intact syndesmosis and were CT scanned at a slice thickness of 0.63 mm (SOMATOM Emotion 6; Siemens Healthcare GmbH, Erlangen, Germany) under weightbearing (700 N) in five different foot positions: neutral position (NP), 20° dorsal / plantar flexion (DF / PF), and 15° external / internal rotation (ER / IR). Subsequently, all three parts (AiTFL, IOL and PiTFL) of the syndesmotic complex were transected under visual control to simulate an isolated Grade 3 injury according to the West Point Ankle Grading System at each of the 14 ankles [7]. The previously executed CT scans were rerun for the injured ankles in the same five positions.

Subsequently, the tibia and fibula CT data were segmented and exported in Standard Triangle Language (STL) file format using Mimics innovation Suite (Mimics Innovation Suite, Materialise, Leuven, Belgium) as previously described [30]. Evaluation of the STL data sets was performed using an automated script coded in Matlab (MathWorks, Natick, MA, USA) and comprising three major computations.

First, the tibial axis of the ankles was defined by a line connecting the centroids of the tibial cross-sections at a distance of 50 mm and 80 mm proximally to the tibial articular surface and represented the z-axis of the coordinate system for the subsequent evaluations.

Second, in order to evaluate the impact of a torn syndesmotic ligament under axial loading (700 N), each injured tibiofibular dataset was matched with the dataset of the corresponding uninjured ankle in the same foot position. The best fit of matching was based on a rigid iterative closest point algorithm using only tibial geometry. During the matching, each fibula was transformed assuming its relative rigid-body constellation with respect to the corresponding tibia.

**Fig. 2:**
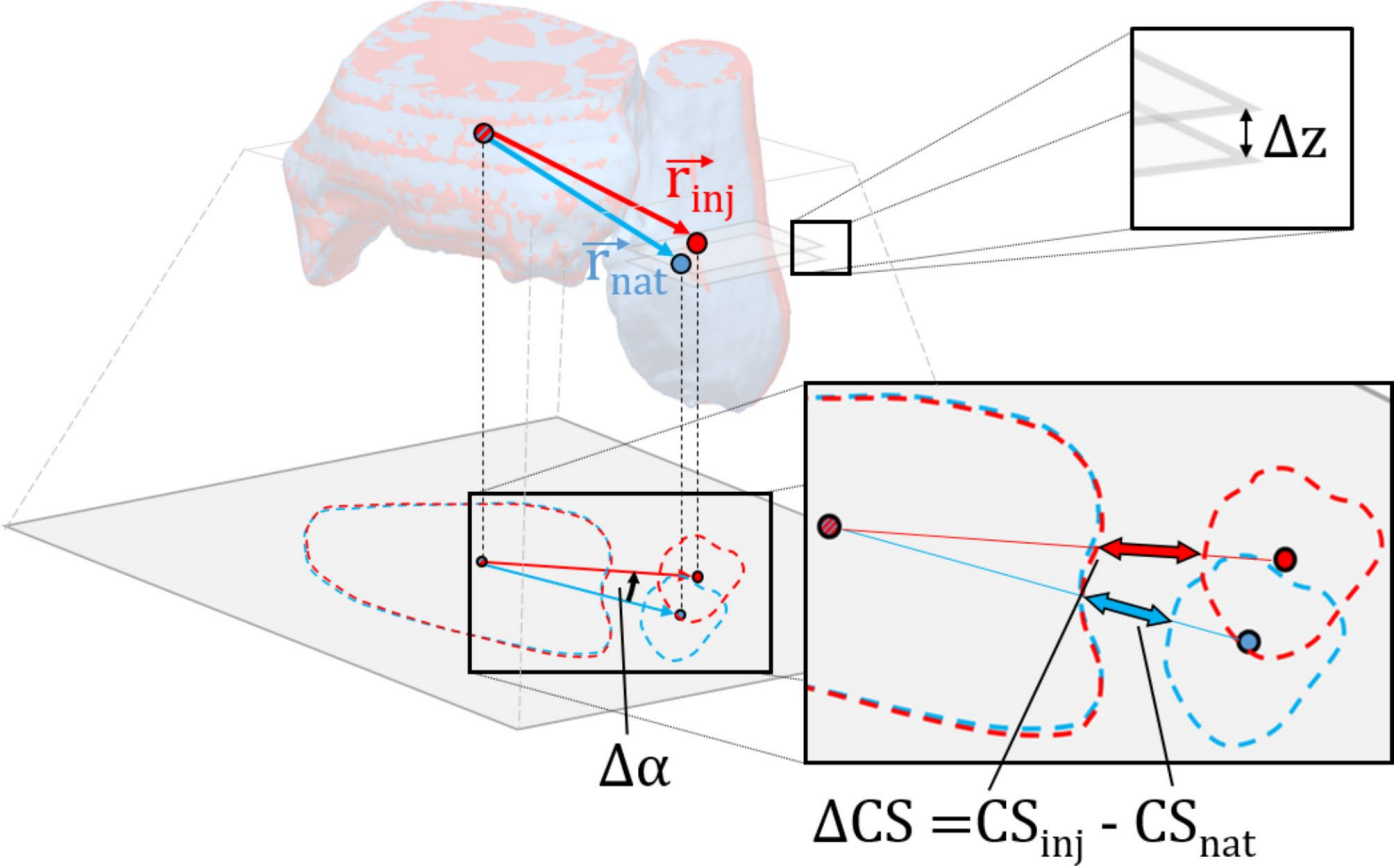
Parameter calculation based on matched DTFJs. One ankle joint with intact syndesmotic ligaments (nat) in external rotation was matched (based only on tibial geometry) to the same ankle joint in external rotation with complete injury to all parts of the syndesmotic ligaments (inj). Clear space difference (∆CS), vertical offset (∆z) and translation angle (∆α) are determined by the vectors connecting the tibial and fibular centroids in each separate state *(*$\:{\overrightarrow{r}}_{nat}$→$\:{\:\overrightarrow{r}}_{inj}$). For calculation of the centers of volume, the tibia and fibula were virtually cut 20 mm proximally to the articular surface. The schematic illustration was made using Geomagic Design X software (3D Systems, Rock Hill, SC, USA) and Microsoft PowerPoint (Microsoft, Redmond, WA, USA)

Third, following matching, differences in the fibular alignment of the uninjured and injured ankle were assessed by calculation of the following three 3D parameters defined in a previous work: clear space (CS, diastasis), translation angle (rotation), and vertical offset (fibular shortening) (Fig. 2) [30, 31]. 

Clear space difference (∆CS) between the injured and uninjured ankle in medio-lateral direction was calculated by measuring the diastasis along the projection of the corresponding connecting vector in a transversal plane (perpendicular to the z-axis) located 10 mm proximally to the articular tibial surface in each separate ankle position [30]. Translation of the fibula in sagittal direction, represented by the translation angle (∆α), was calculated by measuring the angle between the projections of the connecting vectors of tibial and fibular centers of volume for the injured and uninjured ankle in the transversal plane. The translation angle is primarily affected by anteroposterior translation of the fibula, however, medio-lateral translation also slightly influences it [30]. 

The longitudinal change in the fibular position was calculated by measuring the vertical offset (∆z) of the center of volume of both fibulae (along z-axis) [30]. 

Statistical analysis was performed using Matlab. Lilliefors test was run to screen and prove the normality of data distribution, whereas one sample t-test was used to detect significant differences between the states with injured and uninjured syndesmosis. Due to testing of different foot positions, all p-values were adjusted using Holm-Bonferroni correction for multiple comparisons. The level of significance was set to 0.05 for all statistical tests. Post-hoc power analysis was performed by using G*Power (HHU Düsseldorf, Germany).

## Results

**Fig. 3:**
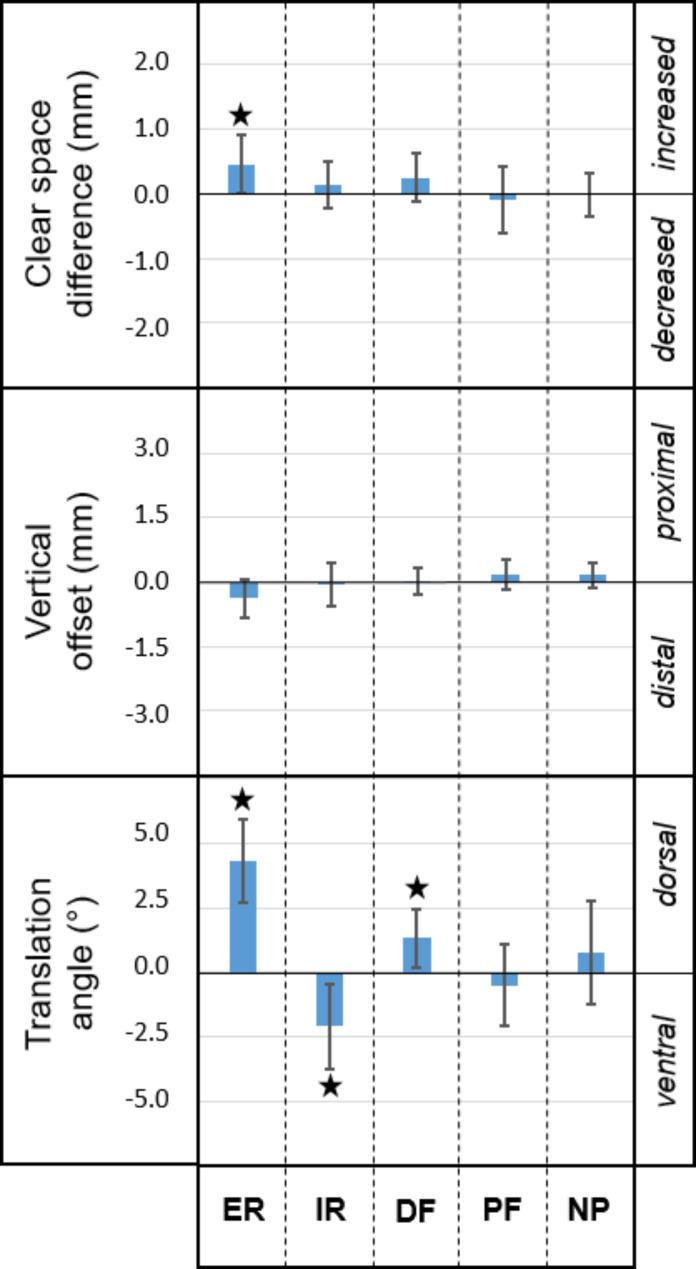
Resulting 3D parameters for each foot position. Clear space difference (∆CS), vertical offset (∆z), and translation angle (∆α) for external/internal rotation (ER/IR), dorsal/plantar flexion (DF/PF) and neutral foot position (NP) in loaded condition (700 N) show changes in configuration of the DTFJ due to the syndesmotic ligament injury (Grade 3 according to West Point Ankle Grading System). Stars indicate values significantly different from zero. Axes are scaled to corresponding thresholds regarding native anatomy (∆CS: ±2 mm, ∆z: ±3 mm, ∆α: ±5°) to visualize better the effect size of syndesmotic ligament injury on the 3D measurements

Clear space difference, translation angle and vertical offset of the injured ankles relative to the corresponding uninjured state are visualized in Fig. 3 for each separate foot position. In addition, their mean values, standard deviations (SDs) and p-values are presented in Table 1.

After transection of all syndesmotic ligaments, no significant differences were detected for clear space (∆CS), fibular translation (∆α), and vertical offset of the fibula (∆z) of the loaded foot in neutral position and plantar flexion (*p* ≥ 0.414).

Transection of the syndesmosis resulted in significant posterior translation of the fibula (1.32°, SD 1.16°, *p* = 0.012) in dorsal flexion. Even larger translation angles due to transection of the syndesmosis were observed in foot rotation. Injury of the syndesmosis led to significant posterior translation in external foot rotation (4.34°, SD 1.63°, *p* < 0.001), and to significant anterior translation in internal foot rotation (-2.08°, SD 1.65°, *p* = 0.006).

In addition, clear space significantly increased after transection of the syndesmosis (0.46 mm, SD 0.46 mm, *p* = 0.029) in external rotation. For external rotation, which was associated with the largest effects, a statistical power greater than 0.90 was calculated for for all three parameters.

**Table 1:** Clear space (CS) difference, vertical offset, and translation angle of injured syndesmotic ligaments relative to the intact state for external/internal rotation (ER/IR), dorsal/plantar flexion (DF/PF) and neutral foot position (NP) in loaded condition (700 N). P-values were adjusted using Bonferroni-Holm correction for multiple comparisons

Foot position	CS difference (mm)			Vertical offset (mm)			Translation angle (°)			
	mean	SD	p	mean	SD	p	mean	SD	p	
ER	0.46	0.46	0.029	− 0.37	0.45	0.094	4.34	1.63	< 0.001	
IR	0.14	0.36	> 0.999	− 0.07	0.50	> 0.999	-2.08	1.65	0.006	
DF	0.25	0.38	0.284	0.00	0.31	> 0.999	1.32	1.16	0.012	
PF	− 0.09	0.51	> 0.999	0.18	0.35	0.579	-0.50	1.57	> 0.999	
NP	− 0.02	0.34	> 0.999	0.17	0.28	0.414	0.75	1.99	> 0.999	

## Discussion

By comparing CT images of uninjured and injured ankles in five different foot positions under axial loading, three main important points were identified in the current study as follows.

1) Controlled internal or external rotational stress results in sagittal instability of the DTFJ in case of unstable syndesmotic lesion;

2) In case of unstable syndesmotic lesion, sagittal instability (anteroposterior translation of the fibula) is more pronounced than transversal instability (diastasis) in all different foot positions;

3) In case of unstable syndesmotic lesion, controlled external rotation of the foot is the most responsive position to detect instability of the DTFJ due to significant changes in diastasis and sagittal translation.

**Fig. 4:**
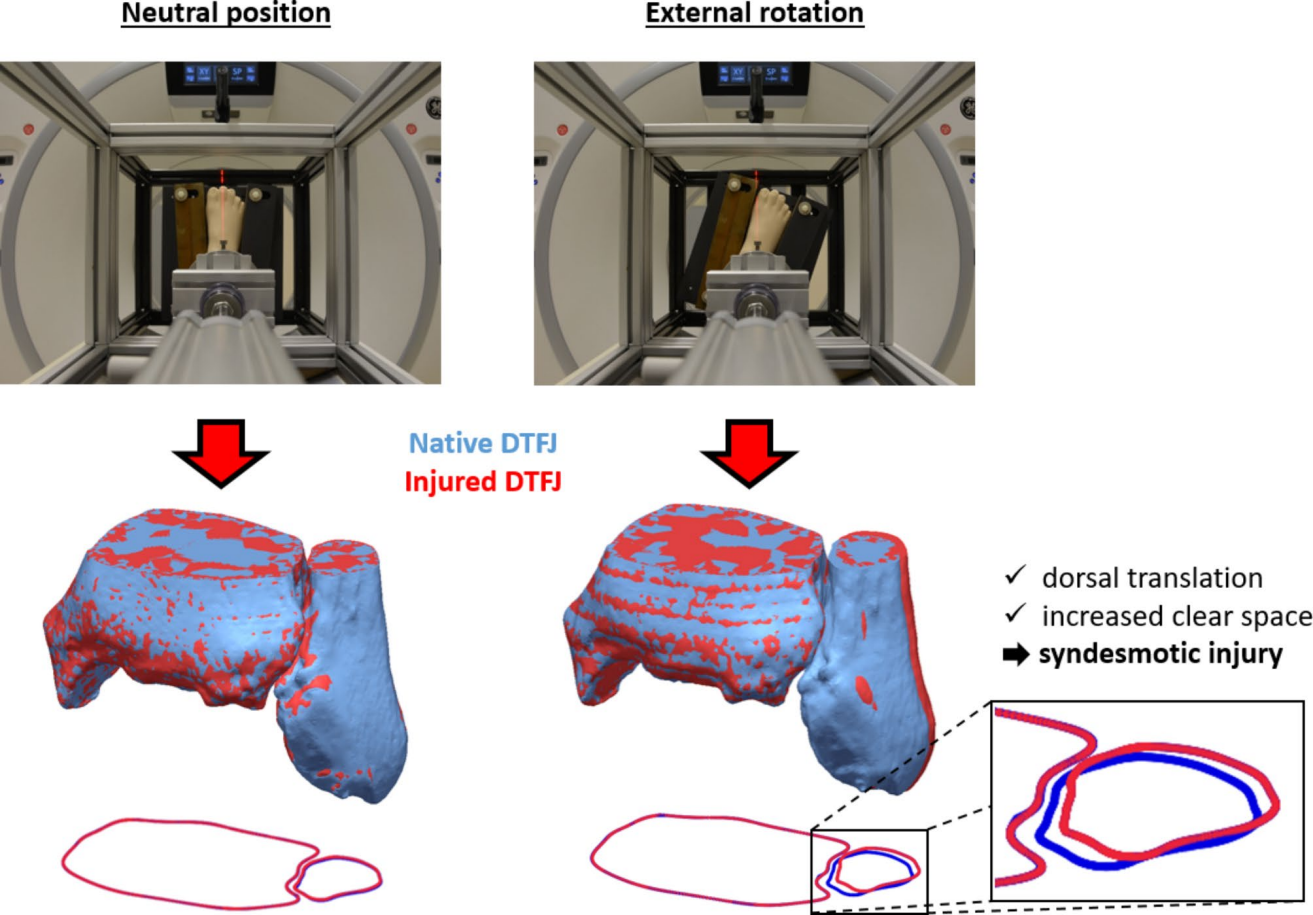
Differences in detection of unstable syndesmotic injuries between neutral foot position and external rotation. Left) Visualization of the neutral foot positioning in the CT frame for a native ankle and the corresponding injured unstable ankle after transection of all syndesmotic ligaments (top) together with the corresponding 3D bony configuration of the DTFJ after best-fit matching of the bony tibial geometry (middle) and the configuration of the unstable injured (red) DTFJ as compared to the native (blue) DTFJ and revealing minimal differences (bottom). Right) Visualization of the foot positioning of the same specimen in 15° external rotation for a native ankle and the corresponding injured unstable ankle after transection of all syndesmotic ligaments (top) together with the corresponding 3D bony configuration of the DTFJ after best-fit matching of the bony tibial geometry (middle) and the configuration of the unstable injured (red) DTFJ as compared to the native (blue) DTFJ and revealing significant differences (bottom)

In contrast to the classification of isolated syndesmotic injuries according to the modified West Point Ankle Grading System, no consensus about a non-invasive diagnostic algorithm exists [7, 32, 33]. It is challenging to differentiate and objectify a stable from an unstable syndesmotic ligament injury, especially in case of Grade IIA and IIB lesions [7, 33]. Current treatment decisions are based on clinical examination, plain radiographs and MRI findings [1, 9, 32, 33]. The consensus of the “European Society of Sports, Traumatology, Knee Surgery & Arthroscopy- Ankle and Foot Associates (ESSKA-AFAS)” recommends assessment in radiographic imaging by measuring tibiofibular clear space, medial clear space, overlap, tibial width and fibular width [7]. All these parameters assess the transversal instability of the DTFJ and do not correlate with findings of syndesmotic ligament injuries on MRI [34]. Especially in cases of partial rupture, radiographs have a low sensitivity for detection [15]. Contrary, the results of the presented 3D analysis demonstrate that an isolated unstable syndesmotic lesion results in a rather more sagittal oriented instability than in the so far investigated transversal one [24]. In the current study, the bidirectional transversal and sagittal instability resulting from a complete unstable syndesmotic lesion was most evident under external rotational stress and featured both a posterior translation of the fibula and an increased clear space (Fig. 4).

Moreover, there is no consensus on the position of the foot during the imaging examinations [7]. Recent studies demonstrated that the position of the foot causes a physiological movement of the fibula relative to the tibia in intact ankles [23]. Specifically, external foot rotation resulted in significant sagittal translation of the fibula in intact ligament conditions [23]. Remarkably, in the present study the sagittal instability of the DTFJ, which could not be detected in neutral position, was more evident under external (4.34°, *p* < 0.001) than under internal (2.08°, *p* = 0.006) rotational stress. The summation of external and internal rotational stress demonstrated a pronounced sagittal instability after complete unstable lesion of the syndesmotic ligaments by an average of 6.42° when compared to the intact condition. This aspect is poorly considered in the assessment of the DTFJ under stress radiography and may pose an explanation for the rate of overlooked instability [24]. Therefore, despite the recommendations of the ESSKA-AFAS – not recommending stress radiography in general – cross-section imaging with the foot under external rotational stress may improve the diagnosis with respect of the bidirectional instability [7, 23, 24]. The benefit of external rotational stress imaging was also confirmed in another human cadaveric study, where transversal instability was detected for partial syndesmotic lesions (AiTFL and IOL) [24]. It is also remarkable that only in external rotation a widening of the clear space was observed, which is currently only considered under external rotation stress test with fluoroscopy as shown in both a systematic review and a cadaveric study by using portable ultrasonography for evaluation of suspected syndesmotic instability [13, 35]. However, sagittal instability is not considered either.

Actually, MRI is the non-invasive gold standard for detection of syndesmotic injuries. However, it represents is a static examination in neutral position, which disregards the effects reported in the presented study under application of rotational stress. Despite its high specificity (95%), this is the main limitation of MRI imaging [7, 12, 17, 36]. It can be hypothesized that MRI examinations for syndesmotic injuries might be more effective if they are performed in external rotated foot positioning.

Large interindividual differences in the anatomy and physiological motion should be considered in the diagnosis of DTFJ instability [23, 25, 26, 31, 37]. Due to this variability, it is difficult to establish absolute values defining instability or physiological congruity. Therefore, bilateral CT imaging is recommended to verify congruity [25, 38]. Furthermore, experience can be adapted from other specialties. For example, when analyzing distal radioulnar joint instability in ambiguous situations, a bilateral CT is performed in pronation, supination, and neutral position. Application of the presented algorithm by using bilateral stress CT examinations may improve diagnostic accuracy and help preoperative decision making.

Based on the results of this study, diagnostic accuracy may be improved by bilateral imaging of both feet in external rotation, followed by comparing the configuration of both DTFJs. Posterior fibular translation and increased clear space highly indicate isolated unstable syndesmotic instability (Fig. 4).

Using the presented standardized segmentation protocol to create 3D models and 3D measurements without rater-dependent anatomical landmarks is a strength of this study. Thus, the measurements become repeatable and independent from the examiner. Furthermore, compared to previous studies with plain radiography, the 3D imaging has the advantage of providing a multidirectional analysis of the joint position in relation to the foot positioning.

Despite the advantages, this 3D study is not without limitations. As in all cadaveric studies, only a limited number of subjects were used. However, due to the large effect size for external rotation, a power greater than 0.9 was achieved for all three parameters despite the limited number of subjects. Furthermore, effects related to the absence of muscle activity cannot be excluded. Nevertheless, the study design represents optimal laboratory simulation comparable to in vivo conditions, as the ankle joint is primarily stabilized by ligaments and the joint capsule. In pre- and postoperative analyses, the patient lies relaxed on the CT examination table, and intraoperatively, muscle activity is minimized by the use of anesthesia and muscle relaxants. Also, the measurement results cannot be related to clinical symptoms. It is also unclear how much rotation of the foot must be considered to measure the maximum extent of instability. The used degrees of rotation were based on the Frick external rotation test [14, 39, 40]. A systematic literature search and meta-analysis demonstrated that the ERST enables differentiation between single-, double- and triple-band injuries of the syndesmal complex under image converter control. It is recommended that the examination be performed bilaterally, with the uninjured opposite side serving as a control. It is remarkable that the demonstrated instability in this study was already evident in 15° rotation of the foot. Comparable studies examined an external rotation of 15–20° [24]. Moreover, in contrast to this study, in clinical practice imaging (CT, MRI) is performed without weightbearing. In this regard, it has been reported that axial weightbearing in different rotational positions of the foot has almost no effect on the alignment of the non-injured and the injured DTFJ [23, 24]. With improved visualization of bony structures via MRI, cost effectiveness and shorter examination times, the observed effects of foot positioning could also find application in this imaging modality. The used automated 3D method is still quite new and not yet widely applied in routine clinical practice. However, this 3D analysis is based on widely available CT imaging, demonstrating a good correlation between the 3D and 2D parameters [31]. Up to date, there are no reliable cut-off values for sagittal and transversal instability. With increasing application of this bilateral examination, these values can be determined.

## Conclusion

External rotation of the foot is the most responsive position for detection of isolated syndesmotic instability. Sagittal instability and increased diastasis resulting from a complete isolated unstable syndesmotic lesion were most evident under external rotational stress. Based on the results, bilateral loaded CT imaging (weightbearing CT) in external rotation of the foot can substantially improve the diagnosis of isolated unstable syndesmotic injuries.

## Data Availability

No datasets were generated or analysed during the current study.

## Abbreviations

∆α: translation angle
∆CS: Clear space difference
∆z: vertical offset
3D: threedimensional
AiTFL: anterior inferior tibiofibular ligament
CS: Clear space
CT: Computer tomography
DF/ PF: dorsal flexion/ plantar flexion
DTFJ: distal tibiofibular joint
ER/ IR: external rotation/ internal rotation
IOL: interosseous membrane/ligament
MRI: magnetic resonance imaging
mm: Millimeter
N: Newton
NP: neutral position
PiTFL: posterior inferior tibiofibular ligament
PMMA: Polymethylmethacrylate
SD: Standard Deviation
STL: Standard Triangle Language
WBCT: weightbearing cone-beam computed tomography
